# Functional conservation of RecQ helicase BLM between humans and *Drosophila melanogaster*

**DOI:** 10.1038/s41598-019-54101-5

**Published:** 2019-11-26

**Authors:** Rebecca L. Cox, Carolyn M. Hofley, Pallavi Tatapudy, Romil K. Patel, Yaron Dayani, Madison Betcher, Jeannine R. LaRocque

**Affiliations:** Department of Human Science, Georgetown University Medical Center, Washington, DC 20057 USA

**Keywords:** Genomic instability, Double-strand DNA breaks

## Abstract

RecQ helicases are a family of proteins involved in maintaining genome integrity with functions in DNA repair, recombination, and replication. The human RecQ helicase family consists of five helicases: BLM, WRN, RECQL, RECQL4, and RECQL5. Inherited mutations in RecQ helicases result in Bloom Syndrome (*BLM* mutation), Werner Syndrome (*WRN* mutation), Rothmund-Thomson Syndrome (*RECQL4* mutation), and other genetic diseases, including cancer. The RecQ helicase family is evolutionarily conserved, as *Drosophila melanogaster* have three family members: DmBlm, DmRecQL4, and DmRecQL5 and DmWRNexo, which contains a conserved exonuclease domain. DmBlm has functional similarities to human BLM (hBLM) as mutants demonstrate increased sensitivity to ionizing radiation (IR) and a decrease in DNA double-strand break (DSB) repair. To determine the extent of functional conservation of RecQ helicases, *hBLM* was expressed in *Drosophila* using the GAL4 > *UASp* system to determine if GAL4 > *UASp::hBLM* can rescue *DmBlm* mutant sensitivity to IR. *hBLM* was able to rescue female *DmBlm* mutant sensitivity to IR, supporting functional conservation. This functional conservation is specific to BLM, as human GAL4 > *UASp::RECQL* was not able to rescue *DmBlm* mutant sensitivity to IR. These results demonstrate the conserved role of BLM in maintaining the genome while reinforcing the applicability of using *Drosophila* as a model system to study Bloom Syndrome.

## Introduction

One of the fundamental biological processes of the cell is to transmit genetic information to its daughter cells efficiently and accurately. Loss of genome integrity may cause detrimental changes, including mutations and other rearrangements, that can lead to cell death in single-cell organisms or tumorigenesis in multicellular organisms. Maintaining genome integrity at both the cellular and organismal level is important as genome instability is a hallmark of human diseases associated with cancer, premature aging, and developmental defects. One factor that may result in genome instability is DNA damage. There are multiple types of DNA damage, including inter and intrastrand crosslinks, base-pair mutations, and single- and double-strand breaks (DSBs)^1^.

**Figure 1:**
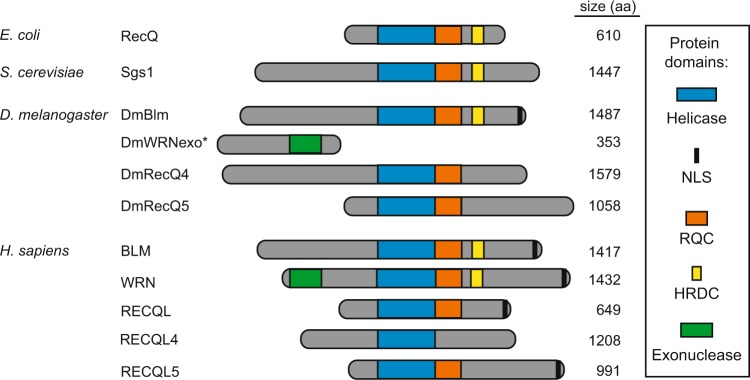
RecQ helicase family. Schematic representation of the RecQ helicase protein family within and across multiple species. The highly conserved RecQ helicase superfamily domains (blue) align all protein schematics, and functionally relevant motifs or stretches of amino acid acids are colored as indicated (not to scale). *DmWRNexo lacks the RecQ helicase domain and is not considered a RecQ helicase, but included for illustrative purposes. NLS, Nuclear Localization Signal; RQC, RecQ C-terminal; HRDC, Helicase and RNaseD C-terminal. Protein lengths (amino acids) are provided.

One family of proteins important for repairing DSBs are RecQ helicases, which are identified through their conserved RecQ helicase superfamily domain^2^. RecQ helicases are involved in maintenance of genome integrity, specifically through DNA repair of DSBs and DNA damage that occurs during DNA replication^3,4^. The human RecQ helicase family consists of five helicases: BLM, WRN, RECQL, RECQL4, and RECQL5. Inherited mutations in RecQ helicases result in several genetic diseases, including Bloom Syndrome (mutations in *BLM*), Werner Syndrome (mutations in *WRN*), Rothmund-Thomson Syndrome (mutations in *RECQL4*), and other diseases, including cancer^5^. While loss of RecQ helicases leads to genome instability, the importance of these proteins is highlighted by their conservation across many species. All model organisms have at least one RecQ helicase, from single-celled prokaryotes to multicellular eukaryotes^6^ (Figs. 1 and S1). For example, *Drosophila melanogaster* has three RecQ helicases: DmBlm, DmRecQL4, and DmRecQL5 and an ortholog of the human WRN exonuclease domain, DmWRNexo^7^.

A well-characterized RecQ helicase found in many eumetazoans is BLM (Figs. 1 and S1). Loss of BLM helicase function in humans results in Bloom Syndrome (BS), a rare autosomal recessive disease. Clinical manifestations of the disease include short stature, male infertility, and predisposition to all forms of cancer due to the high increase in chromosome instability^8,9^. BLM is involved in several aspects of the DSB repair pathway called homologous recombination (HR) including 5′ to 3′ end resection^10,11^, branch migration of the D loop^12^, and dissolution of double Holliday junctions by decatenation^13–15^. Mutations in BLM result in chromatid gaps and breaks, chromosome rearrangements, and an increase in sister chromatid exchanges^16,17^.

These characteristics and deficiencies seen in BS patients and cells demonstrate chromosome instability, which may also be reflected in hypersensitivity to DNA-damaging agents. Supporting this, there is an increase in hypersensitivity of both human BS cells and *Drosophila Blm* mutants to ionizing radiation (IR)^18,19^. Moreover, *BLM* orthologs have similar roles in both organisms based on biochemical^20^ and genetic experiments^21,22^. The two BLM orthologs also share similar protein domains (Fig. 1), consensus in the RecQ helicase domain (Supplementary Fig. S2A)^23^, and 30% identity and 47% similarity across the entire protein sequence^24^. These observations prompted us to investigate the extent of functional conservation of BLM between these humans and *Drosophila*. Specifically, the ability of BLM to repair IR-induced DSBs was tested by examining the sensitivity of *DmBlm* mutants to IR in the presence of *hBLM*.

Additionally, based on the conservation of RecQ helicase domain within species (Supplementary Figs. S1 and S2B), and the presence of RECQL in humans (hRECQL; Fig. 1), we determined whether hRECQL shares similar functions to repair IR-induced damage. This was tested by investigating whether hRECQL could rescue IR sensitivity in *DmBlm* mutants. Taken together, analyzing functional conservation of both BLM and RECQL can provide insights into evolutionary patterns of RecQ helicases.

## Results

### GAL4 > *UASp* system is effective in rescuing *DmBlm*^*N1*^ mutant IR sensitivity by wild-type *DmBlm* expression

**Figure 2:**
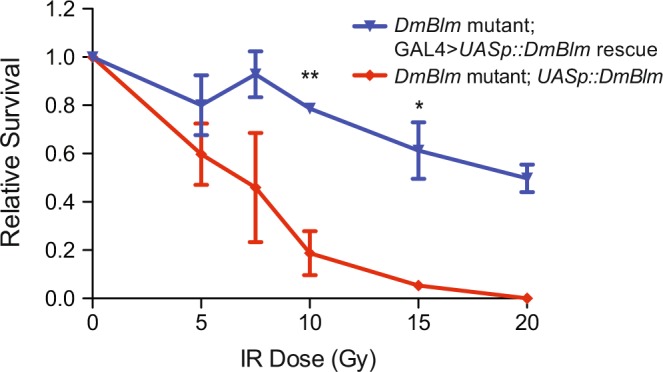
*DmBlm* rescue of *DmBlm*^*N1*^ mutant IR sensitivity. *DmBlm*^*N1*^ heterozygotes carrying *UASp::DmBlm* transgene were crossed to *DmBlm*^*N1*^ heterozygotes carrying the *Act5c::GAL4* transgene. Larvae progeny of this cross were exposed to gamma irradiation up to 20 Gy. Survival to adulthood of *UASp::DmBlm* homozygous *DmBlm*^*N1*^ mutants, relative to survival of all flies, is shown for mutants with (rescue, blue) or without (mutant, red) *Act5c::GAL4*. *p < 0.05, **p < 0.01, two-tailed unpaired Student’s t-Test. Means and standard errors of the mean of 2–4 replicates from two different experiments are shown.

One of the benefits of the GAL4 > *UAS* system is the ability to express a gene of interest both spatially and temporally, depending on the GAL4 driver as well as the UAS sequence associated with a gene of interest^25^. One of the first GAL4 > *UAS* systems developed utilized the UASt sequence, which results in expression in somatic cells of males and females^26^. However, considering DmBlm plays important roles in both mitotic and meiotic recombination in *Drosophila*^19,22,27–30^, additional expression in the female germline was also established using the GAL4 > *UASp* system, where GAL4 drivers were able to express *UASp* gene fusions in the female germline, as well as male and female somatic cells^31,32^. To confirm that GAL4 > *UASp* system could express the RECQ genes of interest at levels capable of rescuing DmBlm mutant phenotypes, IR sensitivity was measured in *DmBlm*^*N1*^ null mutants^19^ with and without GAL4 > *UASp::DmBlm* expression. *DmBlm*^*N1*^ mutants with GAL4* > UASp::DmBlm* expression had significantly greater survival at 10 and 15 Gy than *DmBlm*^*N1*^ mutants without GAL4 expression (Fig. 2; p < 0.01, 0.05 respectively; two-tailed unpaired Student’s t-Test), confirming that the GAL4 > *UASp* expression system was sufficient to rescue IR sensitivity in both males and females (Supplementary Fig. S3A,B).

### *hBLM* expression rescues *DmBlm*^*N1*^ mutant female IR sensitivity

**Figure 3:**
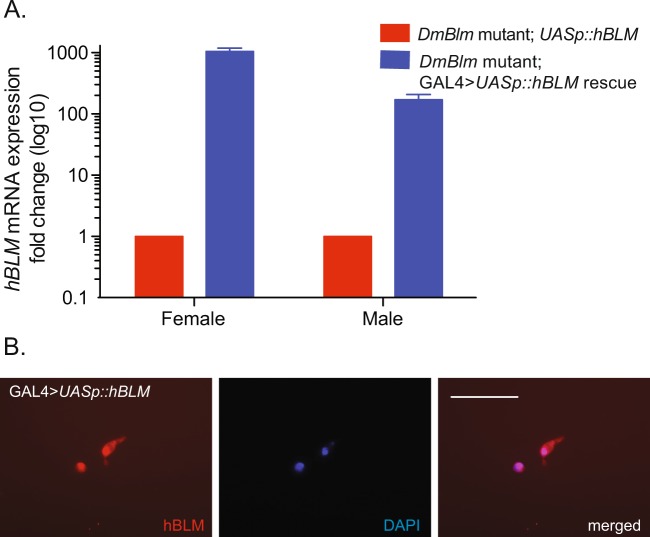
hBLM expression and localization. (**A**) Flies with *Act5c::GAL4* and *UASp::hBLM* transgenes (blue) showed greater *hBLM* mRNA expression than baseline levels without the *GAL4* > *UASp* expression system (red). Mean fold change and standard errors of the mean are shown. (**B**) *UASp::hBLM* was co-transfected with *Act5c::GAL4* into S2 *Drosophila* cells. Localizing to the nucleus (stained with DAPI) was evident in transfected cells using hBLM-specific immunofluorescence. Scale bar is 32 µm.

Considering the strong sequence conservation between DmBlm and hBLM as well as the similar roles these proteins play in genome maintenance, it was determined whether there is functional conservation of BLM between *Drosophila* and humans. To determine if hBLM could rescue DmBlm mutant phenotypes, a GAL4 > *UASp::hBLM* system was established. To validate the effectiveness of our system, the expression of hBLM mRNA was measured. Females with both *Act5c::GAL4* and *UASp::hBLM* showed over 1000-fold increase of *hBLM* mRNA expression compared to females with only the *UASp::hBLM* transgene (Fig. 3A; 1042.9 +/− 139.7-fold increase; p < 0.0001, unpaired Student’s t-Test). Males with both *Act5c::GAL4* and *UASp::hBLM* transgenes showed over 100-fold increase of *hBLM* mRNA expression compared to *UASp::hBLM* males (Fig. 3A; 170.5 +/− 37.5-fold increase; p < 0.01, unpaired Student’s t-Test). Differences in expression between females and males was significant (p < 0.01, two-tailed unpaired Student’s t-Test).

After confirming the GAL4 > *UASp* system could increase expression levels in the whole organism, nuclear localization, a requirement for accurate IR-induced DSB DNA repair, was analyzed. *UASp::hBLM* was transiently transfected into *Drosophila* S2 cells with and without *Act5c::GAL4* expression vector. hBLM localized to the DAPI-stained nucleus in the presence of GAL4 (Fig. 3B).

**Figure 4:**
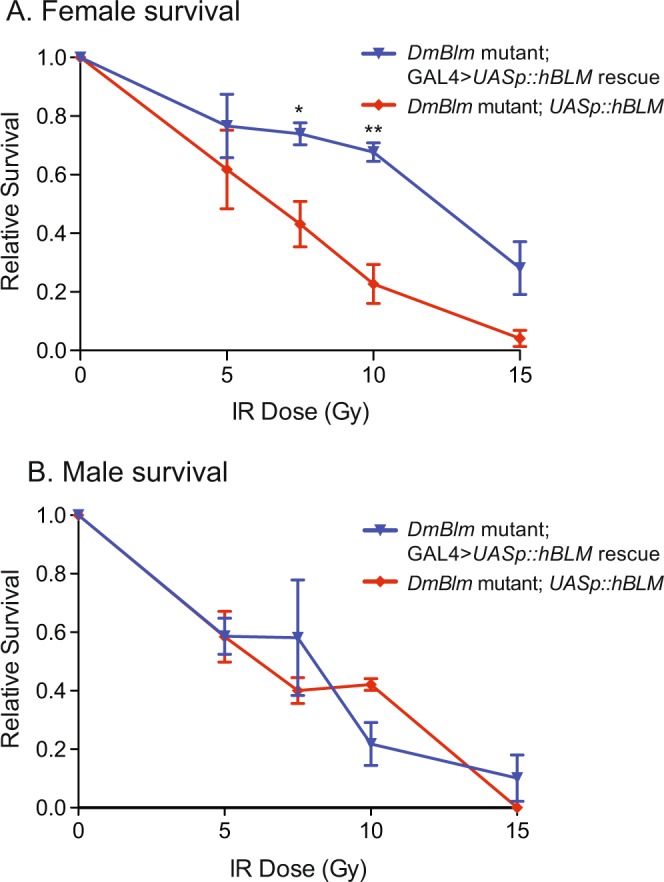
*hBLM* rescue of *DmBlm* mutant IR sensitivity. *DmBlm*^*N1*^ heterozygotes carrying *UASp::hBLM* transgene were crossed to *DmBlm*^*N1*^ heterozygotes carrying the *Act5c::GAL4* transgene. Larvae progeny of this cross were exposed to gamma irradiation up to 15 Gy. Survival to adulthood of *UASp::hBLM* homozygous *DmBlm*^*N1*^ mutants, relative to survival of all flies, is shown for mutants with (rescue, blue) or without (mutant, red) *Act5c::GAL4*. (**A**) *DmBlm*^*N1*^ *UASp::hBLM* females with GAL4 > *UASp::hBLM* expression had significantly greater survival than *DmBlm*^*N1*^ mutants without GAL4 expression at 7.5 and 10 Gy. *p < 0.05, **p < 0.01, two-tailed unpaired Student’s t-Test. (**B**) Both the *DmBlm*^*N1*^ mutant *UASp::hBLM* males and *DmBlm*^*N1*^ mutant *UASp::hBLM* males expressing GAL4 showed similar sensitivity to IR (p > 0.05 for all IR doses, two-tailed unpaired Student’s t-Test). Means and standard errors of the mean of four replicates from two different experiments are shown.

To determine if hBLM maintained functional conservation with DmBlm, *Drosophila* containing *UASp::hBLM* with or without *Act5c::GAL4* transgenes were irradiated at varying IR doses and survivors were quantified. *DmBlm*^*N1*^ mutant females with GAL4 > *UASp::hBLM* expression had greater survival than *DmBlm*^*N1*^ mutant females without GAL4 expression at 7.5 and 10 Gy (p < 0.05 and p < 0.01, respectively; two-tailed unpaired Student’s t-Test; Fig. 4A). *DmBlm*^*N1*^ mutant males with GAL4 > *UASp::hBLM* expression did not have a statistically significant difference in relative survival than *DmBlm*^*N1*^ mutant males without GAL4 expression (Fig. 4B; p > 0.05 at all doses; two-tailed unpaired Student’s t-Test).

### hRECQL expression does not rescue *DmBlm*^*N1*^ mutant IR sensitivity

**Figure 5:**
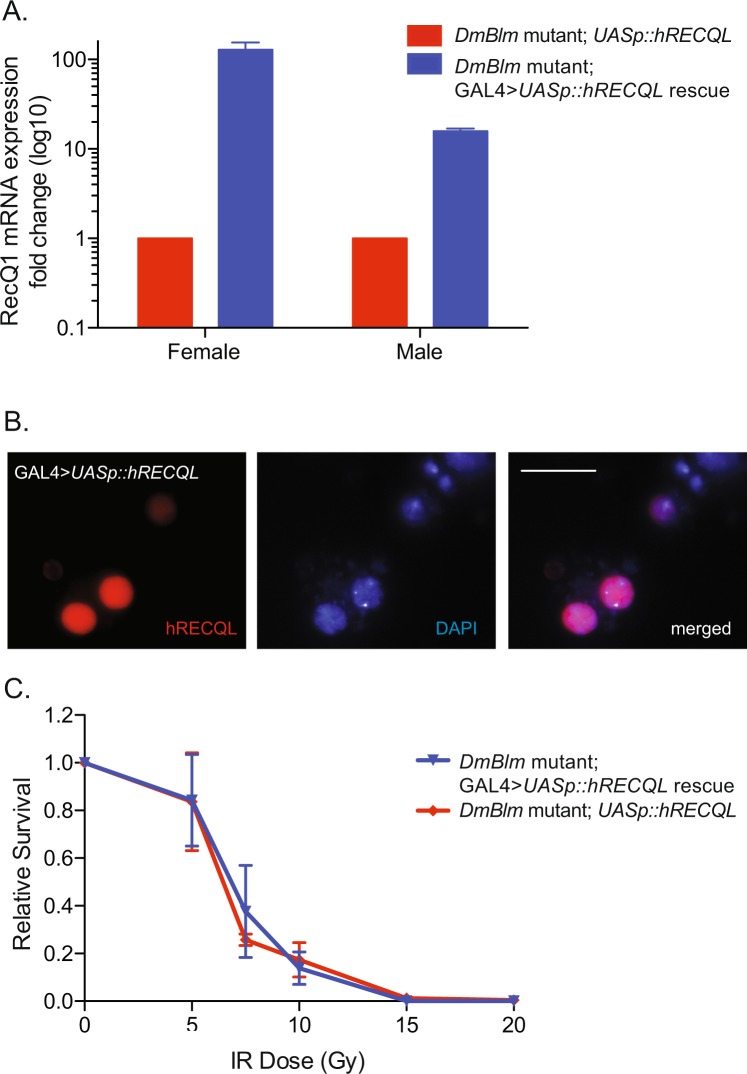
hRECQL expression, localization, and IR sensitivity. (**A**) Flies with *Act5c::GAL4* and *UASp::hRECQL* transgenes (blue) showed greater *hRECQL* mRNA expression than baseline levels without the GAL4 > *UASp* expression (red). Mean fold change and standard errors of the mean are shown. (**B**) *UASp::hRECQL* was co-transfected with *Act5c::GAL4* in S2 *Drosophila* cells. Localizing to the nucleus (stained with DAPI) was evident in transfected cells using hRECQL-specific immunofluorescence. Scale bar is 20 µm. (**C**) *DmBlm*^*N1*^ heterozygotes carrying *UASp::hRECQL* transgene were crossed to *DmBlm*^*N1*^ heterozygotes carrying the *Act5c::GAL4* transgene. Larvae progeny of this cross were exposed to gamma irradiation up to 20 Gy. Survival to adulthood of *UASp::hRECQL* homozygous *DmBlm*^*N1*^ mutants, relative to survival of all flies, is shown for mutants with (rescue, blue) or without (mutant, red) *Act5c::GAL4* (p > 0.05 for all IR doses, two-tailed unpaired Student’s t-Test). Error bars indicate standard errors of the mean from the average survival of three to six replicates from two different experiments.

Considering the rescue of *DmBlm* mutant female IR-sensitivity, and that RECQL is unique to humans (Fig. 1), we wanted to investigate whether there was functional redundancy between RecQ helicases. Evolutionary analysis suggests that RECQL and BLM may share a common ancestor relative to the other RECQ helicases (Supplementary Fig. S1). Thus, we tested whether hRECQL expression could also rescue *DmBlm*^*N1*^ mutant sensitivity to IR. Relative mRNA expression levels of GAL4 > *UASp::hRECQL* were analyzed in whole flies. Females with both transgenes showed 100-fold increase of *hRECQL* mRNA expression compared to females with only the *UASp::hRECQL* transgene (Fig. 5A; 128.4 +/− 26.0 -fold change). Males with both *Act5c::GAL4* and *UASp::hRECQL* transgenes showed statistically significant fold-change differences in *hRECQL* mRNA expression compared to *UASp::hRECQL* males (Fig. 5A; 15.8 +/− 1.1-fold change; p < 0.05, two-tailed unpaired Student’s t-Test). hRECQL nuclear localization in *Drosophila* S2 cells was tested by measuring overlap with nuclear DAPI staining. *UASp::hRECQL* was transiently transfected into S2 cells with and without *Act5c::GAL4* expression vector. hRECQL localized to the nucleus in the presence of GAL4 (Fig. 5B).

*Drosophila* containing *UASp::hRECQL* with or without *Act5c::GAL4* transgenes were irradiated at varying IR doses and survivors were scored. *DmBlm*^*N1*^ mutants with GAL4 > *UASp::hRECQL* expression did not have a statistically significant difference in relative survival than *DmBlm*^*N1*^ mutants without GAL4 expression (Fig. 5C; p > 0.05 at all doses, two-tailed unpaired Student’s t-Test). This was consistent when analyzing female and male survival (Supplementary Fig. S4).

## Discussion

The RecQ helicase family of proteins contain both common and unique roles in maintaining genome stability during replication and recombination^3,4^. Considering the established roles in the RecQ helicase BLM and sequence similarity within the RecQ helicase domain between species (Supplementary Figs. S1 and S2)^24^, we investigated whether there was also functional conservation of BLM across species. Strikingly, GAL4 > *UASp::hBLM* transgenic expression was able to rescue *DmBlm* mutant hypersensitivity to IR in females despite the expression of hBLM without human RMI1 in this study. Human BLM requires RMI1 to promote BLM-dependent dissolution^15^ and TOP3α to complete the dHJ dissolution by branch migration in humans^33,34^. Additionally, germline mutations in both TOP3α and RMI1 result in Bloom Syndrome-like features^35^. However, while there is a *Drosophila* ortholog of TOP3α (DmTop3α), there is no identified ortholog of RMI1. DmTop3α contains topoisomerase functions to maintain the genome, including DSB repair via homologous recombination^30,36,37^. This suggests that DmTop3α may be functioning with hBLM in this context and that the requirement for RMI1 is either specific to human cells, or that a yet-to-be-identified *Drosophila* ortholog is able to function in the absence of human RMI1 to repair IR-induced damage.

While hBLM was able to rescue IR hypersensitivity in *DmBlm*^*N1*^ mutant females, a rescue in males was not observed. This distinction between sexes is not from a difference in expression levels for males and females of *Actin5c*, the promoter driving *GAL4* expression, or *Rpl*3*2*, the housekeeping gene used in our qPCR analyses^38^. A likely explanation for the sex difference in rescue is due to the observation that the UASp sequence results in higher expression in females than in males in the presence of GAL4^32^. Interestingly, the GAL4 > *UASp* system did not result in differential rescue between sexes with expression of wild-type *DmBlm* (Supplementary Fig. S3A,B), which is consistent with the similar relative fold-increase in expression of *DmBlm* in the presence of GAL4 between sexes (Supplementary Fig. S3C). While factors such as integration site of the *UASp* transgenes could impact expression or mRNA stability, our results indicate that the ability of hBLM to repair IR-induced damage in *Drosophila* may be dose-dependent. Future studies using *UASt* or multiple insertions of the *UASp::hBLM* transgene could test this hypothesis. It is important to note that the GAL4 > *UASp* system in flies may lead to expression levels that differ from endogenous *hBLM* expression levels in humans. Thus, our interpretation of our results is that the GAL4 > *UASp::hBLM* expression can rescue *DmBlm* mutant sensitivity,

An alternative interpretation of our results is that hBLM and DmBlm function differently such that overexpression of hBLM compensates for the *DmBlm* mutant defect. This would posit that in *DmBlm* mutants expressing *hBLM*, IR-induced damage is repaired by an unknown hBLM-dependent and DmBlm-independent pathway. However, we suggest that it is more likely that there is functional conservation between hBLM and DmBlm based on foundational work with model systems. For example, in the budding yeast *Saccharomyces cerevisiae*, mutant phenotypes are rescued with galactose-induced overexpression of human orthologs in almost half of the genes analyzed^39^. Kachroo, *et al*. found that amino acid similarity above 50% was a strong predictor for functional conservation across many pathways^39^. Specific to DNA repair, expression of human *RAD52*, a homologous recombination protein conserved from bacteriophages to humans, rescues homologous recombination defects in *S*. *cerevisiae rad52* mutants^40^. Similarly, functional conservation between humans and *Drosophila* has been determined using similar approaches^41–43^. Considering functional conservation analyses in model systems and the 47% amino acid identity between hBLM and DmBlm^24^, we suggest that functional conservation is the more likely explanation for the results reported here.

Considering the ability of hBLM to rescue *DmBlm* mutant IR sensitivity and the presence of a fifth RecQ helicase in humans, we tested whether hRECQL could also rescue DmBlm mutant sensitivity. Phylogenetic analyses suggest that the additional RECQL family member in humans may be due to duplicate gene evolution which resulted in subfunctionalization (Supplementary Fig. S1), where each duplicated gene, BLM and RECQL, retained different subfunctions of the ancestral gene^44^. To determine if hRECQL maintained functions similar to hBLM, we tested whether hRECQL could rescue *DmBlm* mutant phenotypes similar to hBLM. However, despite conservation of the RecQ helicase domains of DmBlm and hRECQL (Supplementary Fig. S2B) and 36% identity and 54% similarity across the entire protein sequence^24^, expression of GAL4 > *UASp::hRECQL* was not able to rescue *DmBlm* mutant IR hypersensitivity. This suggests that these two human RecQ helicases do not maintain common functions in repairing IR-induced DNA damage and that RECQL and BLM are indeed a result of gene duplication and subfunctionalization from a common ancestor. This is also supported by the well-characterized, distinct roles of RecQ helicase family during replication and recombination^3,4,45,46^. Our data and phylogenetic analyses suggest that hBLM most likely maintains the ancestral gene functions observed in DmBlm, whereas hRECQL maintains functions that are distinct from BLM. However, expression levels must be considered, as relative fold change in expression of *RECQL* in the presence of GAL4 was ~10 times less in males and females compared to *hBLM* transgenic lines.

In conclusion, RECQL has a unique function in humans that is not conserved in *Drosophila* Blm. However, our result that GAL4 > *UASp::hBLM* rescues IR sensitivity of *DmBlm*^*N1*^ null mutant females suggests evolutionary functional conservation of the *BLM* gene between *Drosophila* and humans. These findings support the use of *Drosophila* as a model organism to study Bloom Syndrome due to the functional conservation between cellular pathways. This evolutionary conservation also supports the critical role of BLM in maintaining genome integrity in all cells, thus suggesting why the loss of BLM function in Bloom Syndrome patients results in disease associated with high levels of genome instability.

## Materials and Methods

### *Drosophila* stocks and maintenance

*Drosophila* were maintained on standard Nutri-fly Bloomington Formulation medium (Genesee Scientific) at 25 °C using 12-hour light/dark cycles. The *Galactose responsive transcription factor 4* (*GAL4*) stock contains constitutively and ubiquitously expressed *GAL4* driven by *Actin 5c* promoter (*Act5c::GAL4*) and was a kind gift from Gary Karpen (UC-Berkeley). The stock containing the *DmBlm*^*N1 *^^19^ null allele and *UASp::DmBlm* stock, which expresses *DmBlm* under the regulation of GAL4^47^ were from Jeff Sekelsky (UNC-Chapel Hill). *DmBlm*^*N1*^ homozygous mutants were derived from two stocks backcrossed by several generations. The Upstream Activation Sequence *UASp::hBLM* stock and *UASp::hRECQL* were established in this study.

### *hBLM* expression construct and stock

*hBLM* cDNA from pCAGGS + *hBLM*_R12 (R12 clone from Winfried Edelman) was amplified with the primers 5′ ATCAGATCCGCGGCCGCATGGCTGCTGTTCCTCAA (forward) and 5′ CGACTCTAGAGGATCCGGTTTATGAGAATGCATATGAAGGC (reverse) using the CloneAmp HiFi PCR premix according to the manufacturer’s protocol (Clontech). The PCR oligos contained additional *Not*I (forward) and *Bam*HI (reverse) restriction enzyme sites (underlined) that were used for subcloning. Amplified *hBLM* coding sequence with *Not*I/*Bam*HI ends was inserted into a *Bam*HI/*Not*I linearized, ubiquitously expressed UASp fly expression vector, known as pP{UASp}^31^, using the Takara In-Fusion cloning kit following the manufacturer’s instructions (Clontech). Plasmid-purified pP{*UASp::hBLM}* was sent for injection and mapping (BestGene) into *D*. *melanogaster y w* mutant stock. Ten *w* + G1 transformants were selected to establish balanced lines (BestGene). One line inserted in non-repetitive sequences of Chromosome 3 (ideal for downstream recombination with the *DmBlm*^*N1*^ allele) at locus 70B1 in a gene of unknown function, *CG10133*.

The line that inserted at locus 70B1 was used to establish a recombinant line of *UASp::hBLM* with the *DmBlm*^*N1*^ allele using standard genetic techniques. Briefly, crosses were set up for recombination of *UASp::hBLM* and the *DmBlm*^*N1*^ allele to occur within the female germline. Potential recombinant events in the next generation were isolated. To confirm recombination of the *DmBlm*^*N1*^ allele on the *UASp::hBLM* chromosome, flies were screened for *DmBlm*^*N1*^ using *DmBlm*^*N1*^-specific PCR. Genomic DNA was isolated using Squishing Buffer (10 mM Tris-Cl pH 8.2, 25 mM NaCl) and Proteinase K (10 µg), incubation at 37 °C for 30 minutes, followed by inactivation at 95 °C for five minutes. PCR was performed using SapphireAmp Fast PCR Master Mix (Clontech) and *DmBlm*^*N1*^-specific primers: 5′ TGAAGGGTGGACCGACGGTC (forward) and 5′ GCCAGAATATCCAAGCGGAC (reverse) following the manufacturer’s instructions.

### *hRECQL* expression construct and stock

*hRECQL* cDNA in pCAGGS + hRECQL was amplified with the primers 5′ ATCAGATCCGCGGCCGCTGTGACCGGCGGCTCTAGA (forward) and 5′ CGACTCTAGAGGATCCATTGCTAGCGGCCGCTCGAG (reverse). Amplified *hRECQL* was inserted into ubiquitously expressed pP{UASp}^31^ and integrated into Chromosome *3* (BestGene) as described above for *UASp:hBLM*. The *UASp::hRECQL DmBlm*^*N1*^ recombinant line was established as described above for *UASp::hBLM DmBlm*^*N1*^.

### Ionizing radiation sensitivity assays

IR sensitivity assays were completed as described previously^19^. Recombinant female *DmBlm*^*N1*^ heterozygotes carrying a *UASp::X* (*DmBlm*, *hBLM*, or *hRECQL*) transgene were crossed to male *DmBlm*^*N1*^ heterozygotes carrying the *Act5c::GAL4* transgene. *Drosophila* were transferred every 24 h up to three times within each experiment, which served as experimental replicates. Third instar larvae progeny of this cross were exposed to gamma IR at various doses (0, 5, 7.5, 10, 15, or 20 Gy). Average relative survival for each class was calculated [# surviving *DmBlm*^*N1*^ mutants (with or without *Act5c::GAL4* and *UASp::X*)/total number of surviving flies] at each dose. The average survival rate was then normalized to relative survival at 0 Gy. An average of 207 (±6.9 S.E.M) total flies for each sex and each dose for all experiments were counted as the total number of flies surviving to determine relative survival as described above. Average relative survival at each dose was determined from 2–6 experimental replicates. Significance was determined by two-tailed unpaired Student’s t-test between average relative survival of *UASp::X DmBlm*^*N1*^ mutants without *Act5c::GAL4* compared to average relative survival of *UASp::X DmBlm*^*N1*^ mutants with *Act5c::GAL4* at each IR dose.

### qPCR analysis of *BLM* and *RECQL* transgenes

For *hBLM* and *hRECQL* expression analysis, untreated *DmBlm*^*N1*^ mutant males and females with *UASp::X* transgene with (rescue) or without (mutant) *Act5c::GAL4* from IR sensitivity assays were collected. For *DmBlm* analysis, untreated wild-type males and females with *UASp::DmBlm* with or without *Act5c::GAL4* were collected. Two flies from the isogenic lines were combined to represent one biological replicate and 1–2 biological replicates per sex and genotype were harvested. mRNA was purified by acid guanidinium thiocyanate-phenol-chloroform extraction with TRIzol (Invitrogen) and RNA Clean and Concentration-5 (Zymo Research). Contaminating DNA was removed by DNA-free rDNase I treatment (Invitrogen). Reverse transcription was performed using Applied Biosystems High-Capacity RNA-to-cDNA kit (Thermo Fisher Scientific), and qPCR was completed with the RT^2^ SYBR Green Master Mix (Qiagen), using RT^2^ qPCR Primers for *D*. *melanogaster RpL32* (PPD10569B), *D*. *melanogaster Blm* (PPD08711A), human *RECQL* (PPH14762A), and human *BLM* (PPH02711B) (Qiagen). All qPCR measurements were obtained using the Mic qPCR Cycler and software (Bio Molecular Systems) using technical triplicates for each biological sample. Cq values for each experimental gene were normalized to that of *DmRpL32* (Cq) to determine relative expression. ΔΔCq values were calculated relative to flies without *Act5c::GAL4* (mutant) to determine relative expression fold change (2-ΔΔCq) in the presence of *Act5c::GAL4*.

### Cell culture, transfection, and immunofluorescence

S2 cells were cultured at 28 °C in Schneider medium supplemented with 10% heat-inactivated fetal bovine serum. Cells were plated at a density of 1.7 × 10^6^ cells per well of a 6-well tissue culture plate containing Poly-L-Lysine coated coverslips. Following a three-hour incubation, cells were then transfected with 2500 ng of appropriate DNA constructs using Cellfectin (Invitrogen) as per the manufacturer’s protocol.

For immunofluorescent labeling, S2 cells were fixed two days post-transfection with 4% formaldehyde in PBS for 15 min. Cells were then permeabilized and blocked with 0.1% Triton X-100, 1% BSA in TBS for 1 hour at room temperature. Coverslips were incubated cell side down overnight in a humidified chamber with polyclonal hBLM antibody (Invitrogen PA5-27384; 1:120 dilution), or hRECQL antibody (Santa Cruz, H-110; 1:500 dilution) in blocking solution. The following day, cells were briefly washed in 1X PBS + 0.1% Tween-20 and then incubated in HRP-conjugated secondary antibody at a 1:500 dilution in blocking solution for one hour in the dark (Alexa Fluor 594 donkey anti-rabbit IgG for hBLM and Alexa Fluor 594 goat anti-rabbit IgG for hRECQL). Post-treatment slides were washed briefly, counterstained with DAPI, and mounted with Vectashield (Vector laboratories) on Superfrost Plus slides (Sigma) and sealed with nail polish. Slides were allowed to dry for 24 hours pre-imaging. Histological negative controls included staining of non-transfected cells as well as cells transfected with only UASp or GAL4 expression vectors individually (data not shown). Images were captured within one week of experimental procedure using a fluorescent confocal ZEISS Axiovert 200 m microscope at 40X (for hBLM) or 63X (for hRECQL) magnification.

## Supplementary information

Supplementary Information

## Data Availability

All data generated or analyzed during this study are included in this published article (and its Supplementary Information Files).
