# Supplementary material for: Functional conservation of RecQ helicase BLM between humans and Drosophila melanogaster
Source: Sci Rep. 2019 Nov 26;9:17527. doi: 10.1038/s41598-019-54101-5 (PMC6879748; doi:10.1038/s41598-019-54101-5)
Supplement: Supplementary file 1 — Supplementary Information [file 41598_2019_54101_MOESM1_ESM.pdf]

Supplementary Information

Functional conservation of RecQ helicase BLM between humans and *Drosophila melanogaster*

Rebecca L. Cox[1], Carolyn M. Hofley[1], Pallavi Tatapudy[1], Romil K. Patel[1], Yaron Dayani[1], Madison Betcher[1], and Jeannine R. LaRocque[1,*]

[1]Department of Human Science, Georgetown University Medical Center, Washington DC 20057, USA

Key words: RecQ helicase, RECQL, BLM, *Drosophila melanogaster*

[*]Corresponding author:
Jeannine R. LaRocque
School of Nursing and Health Studies
265 St. Mary's Hall
3700 Reservoir Rd. NW
Washington, DC 20057
Phone: (202)-687-2807; Fax: (202)-687-5553
jan.larocque@georgetown.edu

Supplementary Materials and Methods

Sequence alignments and evolutionary genetic analysis. Alignment of RecQ superfamily domains of hBLM (AAI43281.1), DmBlm (NP_524319.2), and hRECQL (P46063.3) were developed using MultAlign (Corpet, 1988). RecQ superfamily domains were defined by Conserved Domain Database (Marchler-Bauer, et al., 2017).

For molecular phylogenetic analysis, sequences used were selected on the presence of a RecQ superfamily domain as defined by the Conserved Domain Database (Marchler-Bauer, et al., 2017). A total of 28 sequences were analyzed from organisms including: *Escherichia coli* (Ec; bacteria), *Saccharomyces cerevisiae* (Sc; baker's yeast), *Hydra vulgaris* (Hv; fresh water polyp), *Amphimedon queenslandica* (Aq; reef sponge), *Aplysia californica* (Ac; California sea hare), *Drosophila melanogaster* (Dm; fruit fly), *Caenorhabditis elegans* (Ce; nematode), *Danio rerio* (Dr; zebrafish), and *homo sapiens* (Hs; human).

Complete amino acid sequences analyzed (with Accession numbers) were: EcRecQ (YP_026263.3), ScSgs1p (GenBank: AAB60289.1), HvBLM (predicted, XP_002167927.3), HvWRN (predicted, XP_012558515.1), HvRECQL (predicted, XP_002159097.2), AqRECQL (predicted, XP_003385544.1), HsBLM (AAI43281.1), HsWRN (AAC63361.1), HsRECQL (P46063.3), HsRECQL4 (NP_004251.3), HsRECQL5 (XP_005257875.1), DmBlm (NP_524319.2), DmRecQ4 (NP_652607), DmRecQ5 (NP_729983), CeBLM (NP_502390), CeWRN (NP_001022657), CeRECQL, CeRECQL5 (NP_001309439.1), AcBLM (XP_005098478), AcWRN (XP_005102148), AcRECQL (XP_012944528), AcRECQL4 (XP_012935837), AcRECQL5 (XP_012944808), DrBlm (XP_017207538), DrWrn (XP_009302246), DrRecQL (NP_001038561), DrRecQL4 (XP_001920631), DrRecQL5 (XP_005164331).

Alignments and subsequent evolutionary analyses were conducted in MEGA7 (Kumar, et al. 2016). The evolutionary history was inferred by using the Maximum Likelihood method based on the JTT matrix-based model (Jones, et al. 1992). Initial tree for the heuristic search were obtained automatically by applying Neighbor-Join and BioNJ algorithms to a matrix of pairwise distances estimated using a JTT model, and then selecting the topology with superior log likelihood value. All positions containing gaps and missing data were eliminated. There was a total of 74 positions in the final dataset.

Supplementary References

Corpet, F. Multiple sequence alignment with hierarchical clustering. *Nucleic Acids Res.* **16**, 10881-10890 (1988).

Jones D.T., Taylor W.R., and Thornton J.M. The rapid generation of mutation data matrices from protein sequences. *CABIOS.* **8**, 275-282 (1992).

Kumar S., Stecher G., and Tamura K. MEGA7: Molecular Evolutionary Genetics Analysis version 7.0 for bigger datasets. *Mol. Biol. Evol.* **33**, 1870-1874 (2016).

Marchler-Bauer, A. *et al.* CDD/SPARCLE: functional classification of proteins via subfamily domain architectures. *Nucleic Acids Res.* **45**, D200-D203 (2017).

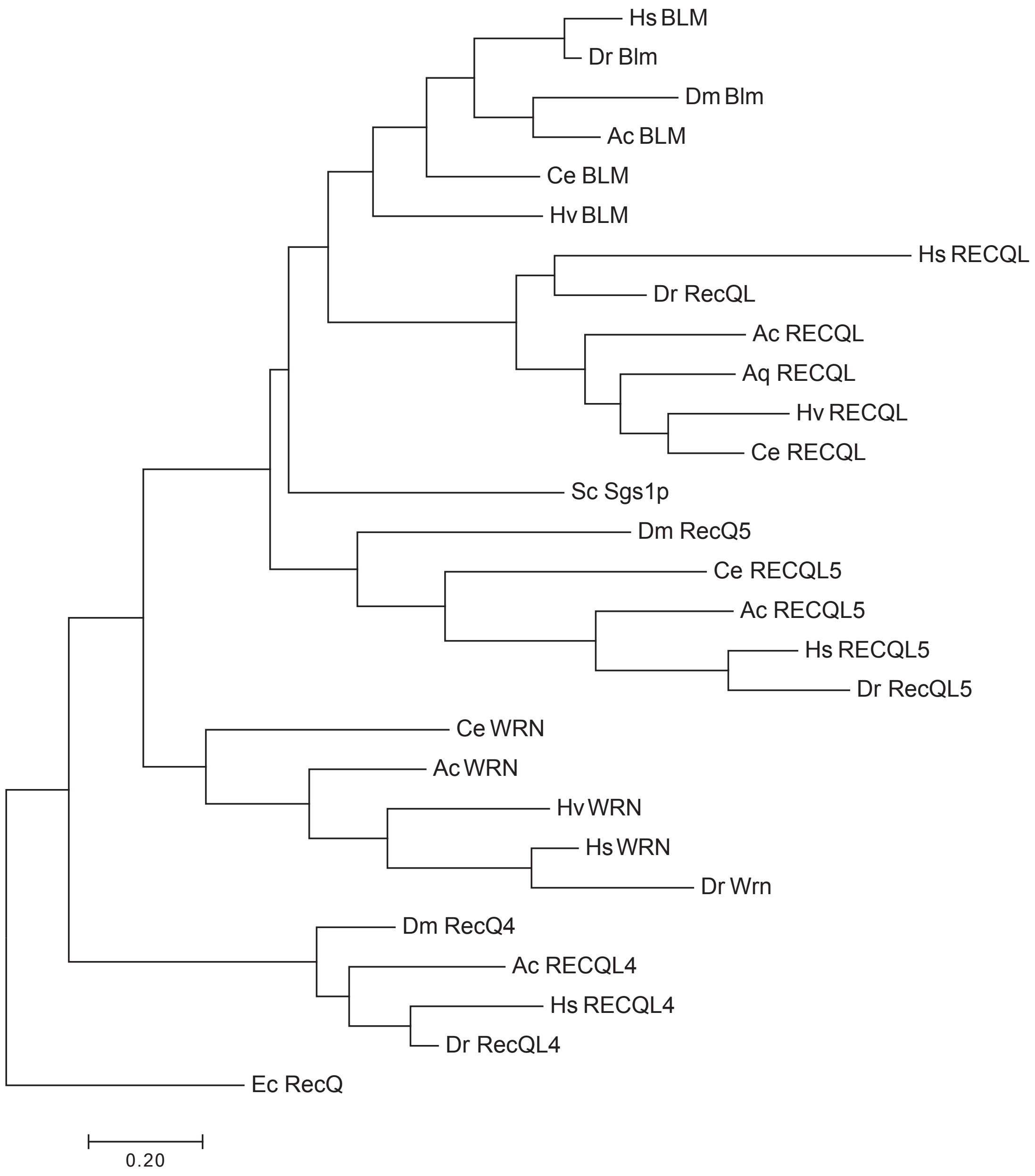

Supplementary Figure S1: Molecular phylogenetic analysis of RecQ family members by Maximum Likelihood method. The evolutionary history was inferred by using the Maximum Likelihood method based on the JTT matrix-based model (Jones, et al. 1992). The tree with the highest log likelihood (-3163.66) is shown. Initial tree(s) for the heuristic search were obtained automatically by applying Neighbor-Join and BioNJ algorithms to a matrix of pairwise distances estimated using a JTT model, and then selecting the topology with superior log likelihood value. The tree is drawn to scale, with branch lengths measured in the number of substitutions per site. Evolutionary analyses were conducted in MEGA7 (Kumar, et al. 2016). The tree is rooted by *Escherichia coli* RecQ (Ec RecQ). Organisms include: *Saccharomyces cerevisea* (Sc; baker's yeast), *Hydra vulgaris* (Hv; fresh water polyp), *Amphimedon queenslandica* (Aq; reef sponge), *Aplysia californica* (Ac; California sea hare), *Drosophila melanogaster* (Dm; fruit fly), *Caenorhabditis elegans* (Ce; nematode), *Danio rerio* (Dr; zebrafish), and *homo sapiens* (Hs; human). DmWRNexo was excluded due to the absence of a RecQ superfamily domain. Accession numbers of the protein sequences used are provided in Supplementary Information Materials and Methods.

A

```
           1       10        20        30        40        50        60        70        80        90       100
           |--------+---------+---------+---------+---------+---------+---------+---------+---------+---------|
     hBLM  KIFHKKFGLHNFRTNQLEAINAALLGEDCFILMPTGGGKSLCYQLPACVSPGVTVVISPLRSLIVDQVQKLTSLDIPATYLTGDKTDSEATNIYLQLSKK
    DmBlm    LSYSFGLKSFRPNQLQVINATLLGNDCFVLMPTGGGKSLCYQLPAILTEGVTIVISPLKSLIFDQINKLASLDICAKSLSGEQKMADVMAIYRDLESQ
Consensus  ..lhkkFGLhnFRpNQL#aINAaLLG#DCF!LMPTGGGKSLCYQLPAclseGVT!VISPLrSLIfDQ!#KLaSLDIcAksLsG#qkda#amaIYr#Lekq

           101     110       120       130       140       150       160       170       180       190      200
           |--------+---------+---------+---------+---------+---------+---------+---------+---------+---------|
     hBLM  DPIIKLLYVTPEKICASNRLISTLENLYERKLLARFVIDEAHCVSQWGHDFRQDYKRMNMLRQKFPSVPVMALTATANPRVQKDILTQLKILRPQVFSMS
    DmBlm  PPMVKLLYVTPEKISSSARFQDTLDTLNSNNYISRFVIDEAHCVSQWGHDFRPDYKKLGVLKKRFPNVPTIALTATATPRVRLDILAQLNLKNCKWFLSS
Consensus  dPi!KLLYVTPEKIcaSaRlqdTL#nLnernliaRFVIDEAHCVSQWGHDFRqDYKr$nmLrqrFPnVPtiALTATAnPRVrlDILaQLnilrcqwFlmS

           201     210       220       230       240       250       260       270       280       290      300
           |--------+---------+---------+---------+---------+---------+---------+---------+---------+---------|
     hBLM  FNRHNLKYYVLPKKPKKVAFDCLEWIRKHHPYDSGIIYCLSRRECDTMADTLQRDGLAALAYHAGLSDSARDEVQQKWINQDGCQVICATIAFGMGIDKP
    DmBlm  FNRSNLRYRVLPKKGVSTLDDISRYIRSKPQHFSGIIYCLSRKECDETSKKMCKDGVRAVSYHAGLTDTDRESRQKDWLTGK-MRVICATVAFGMGIDKP
Consensus  FNRhNLrYrVLPKKgkktadDclrwIRkhhqhdSGIIYCLSRrECDemadk$crDGlaAlaYHAGLsDsaR#erQqdWinqd.crVICAT!AFGMGIDKP

           301     310       320       330       340       350       360       370       380       390      400
           |--------+---------+---------+---------+---------+---------+---------+---------+---------+---------|
     hBLM  DVRFVIHASLPKSVEGYYQESGRAGRDGEISHCLLFYTYHDVTRLKRLIMMEKDGNHHTRETHFNNLYSMVHYCENITECRRIQLLAYFGENGFNPDFCK
    DmBlm  DVRFVLHYSLPKSIEGYYQEAGRAGRDGDVADCILYYNYSDMLRIKKMLDSDKALQYNVKKIHVDNLYRIVGYCENLTDCRRAQQLDYFGEH-FTSEQCL
Consensus  DVRFViHaSLPKS!EGYYQEaGRAGRDG#!adCiL%YnYhDmlRiKr$idm#Kag#hntreiHf#NLYriVgYCENiT#CRRaQqLaYFGEn.Fnp#qCl

           401     410       420       430       440       450       460       470       480       490      500
           |--------+---------+---------+---------+---------+---------+---------+---------+---------+---------|
     hBLM  KHPDVSCDNCCKTKDYKTRDVTDDVKSIVRFVQEHSSSQGMRNIKHVGPSGRFTMNMLVDIFLGSKSAKIQSGIFGKGSAYSRHNAE
    DmBlm  ENRETACDNCINKRAYKAVDALEHARKAARAVKDLCSGRSRFTLLHIADVLKGSKIKKIIDFNHHKTP--HHGVLKDWDKNDVHRLLRKMVIDGFLREDL
Consensus  enr#taCDNCcnkraYKarDal#darkaaRaVq#hcSgrgrrnilH!adsgrgsknkl!ddFnghKsa..qhG!lgdgdandrHrae.............
```

B

```
           1       10        20        30        40        50        60        70        80        90       100
           |--------+---------+---------+---------+---------+---------+---------+---------+---------+---------|
     hBLM  KIFHKKFGLHNFRTNQLEAINAALLGEDCFILMPTGGGKSLCYQLPACVSPGVTVVISPLRSLIVDQVQKLTSLDIPATYLTGDKTDSEATNIYLQLSKK
   hRECQL  DILQNVFKLEKFRPLQLETINVTMAGKEVFLVMPTGGGKSLCYQLPALCSDGFTLVICPLISLMEDQLMVLKQLGISATMLNASSSKEHVKWVHAEMVNK
Consensus  dIlqnkFgLenFRpnQLEaINaa$aGe#cFilMPTGGGKSLCYQLPAccSdGfTlVIcPLrSLieDQlqkLkqLdIpATmLnadksdeeakn!ha#$snK

           101     110       120       130       140       150       160       170       180       190      200
           |--------+---------+---------+---------+---------+---------+---------+---------+---------+---------|
     hBLM  DPIIKLLYVTPEKICASNRLISTLENLYERKLLARFVIDEAHCVSQWGHDFRQDYKRMNMLRQKFPSVPVMALTATANPRVQKDILTQLKILRPQVFSMS
   hRECQL  NSELKLIYVTPEKIAKSKMFMSRLEKAYEARRFTRIAVDEVHCCSQWGHDFRPDYKALGILKRQFPNASLIGLTATATNHVLTDAQKILCIEKCFTFTAS
Consensus  #peiKLiYVTPEKIaaSnrliSrLEnaYEarrlaRia!DEaHCcSQWGHDFRqDYKa$niLrrqFPnapliaLTATAnnrVqkDaqkqLcIercqtFsaS

           201     210       220       230       240       250       260       270       280       290      300
           |--------+---------+---------+---------+---------+---------+---------+---------+---------+---------|
     hBLM  FNRHNLKYYVLPKKPKKVAF--DCLEWIRKHHPYDSGIIYCLSRRECDTMADTLQRDGLAALAYHAGLSDSARDEVQQKWINQDGCQVICATIAFGMGID
   hRECQL  FNRPNLYYEVRQKPSNTEDFIEDIVKLINGRYKGQSGIIYCFSQKDSEQVTVSLQNLGIHAGAYHANLEPEDKTTVHRKW-SANEIQVVVATVAFGMGID
Consensus  FNRhNLkYeVrqKkpnkeaF..DclelIrgrhkg#SGIIYClSrr#c#qmadsLQrdGiaAgAYHAnLedeardeVqrKW.na#ecQV!cAT!AFGMGID

           301     310       320       330       340       350       360       370       380       390      400
           |--------+---------+---------+---------+---------+---------+---------+---------+---------+---------|
     hBLM  KPDVRFVIHASLPKSVEGYYQESGRAGRDGEISHCLLFYTYHDVTRLKRLIMMEKDGNHHTRETHFNNLYSMVHYCENITECRRIQLLAYFGENGFNPDF
   hRECQL  KPDVRFVIHHSMSKSMENYYQESGRAGRDDMKADCILYYGFGDIFRISSMVVMENVGQQ--------KLYEMVSYCQNISKCRRVLMAQHFDEV-WNSEA
Consensus  KPDVRFVIHaS$pKSmEnYYQESGRAGRDdeiadCiL%Yg%gD!fRikr$!mMEndG#q........nLYeMVhYC#NIseCRR!q$aahFdEn.fNp#a

           401     410       420       430       440       450       460       470       480      489
           |--------+---------+---------+---------+---------+---------+---------+---------+--------|
     hBLM  CKKHPDVSCDNCCKTKDYKTRDVTDDVKSIVRFVQEHSSSQGMRNIKHVGPSGRFTMNMLVDIFLGSKSAKIQSGIFGKGSAYSRHNAE
   hRECQL  CNK----MCDNCCKDSAFERKNITEYCRDLIKILKQAEEL-----------NEKLTPLKLIDSWMGKGAAKLRVAGV-VAPTLPREDLE
Consensus  CnK....mCDNCCKdka%err#!T#dcrdi!rilq#aeel...........nerlTmnkL!Dif$GkgaAKirsagf.kapalpRe#aE
```

Supplementary Figure S2: Alignment of RecQ superfamily domains of hBLM and DmBlm (A) and hBLM and hRECQL (B). Amino acids with >90% concensus are shown in red. Amino acids with >50% concensus are in blue. Alignments are developed using MultAlign (Corpet, 1988). RecQ superfamily domains are defined by Conserved Domain Database (Marchler-Bauer A *et al.*, 2017).

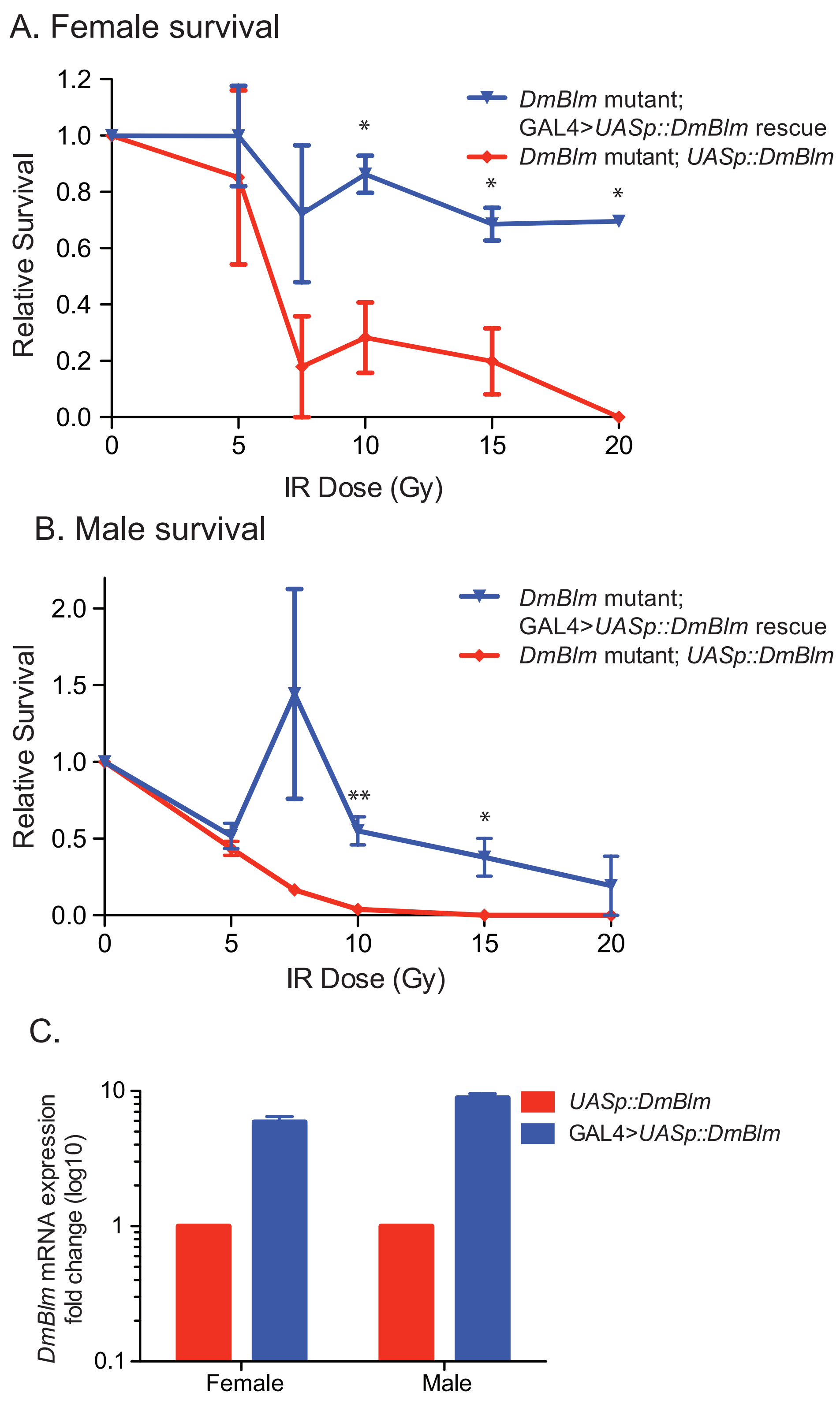

Supplementary Figure S3: Data presented in Figure 2 but separated by female (A) and male (B) survival. (C) Flies with *Act5c::GAL4* and *UASp::DmBlm* transgenes (blue) showed greater *DmBlm* mRNA expression than baseline levels without the GAL4>*UASp* expression system (red). Mean fold change and standard errors of the mean are shown.

A. Female survival

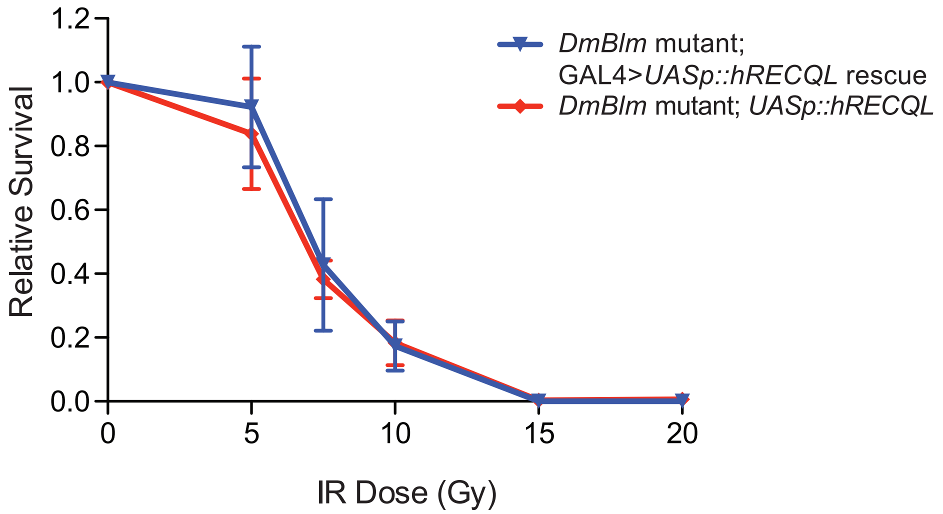

B. Male survival

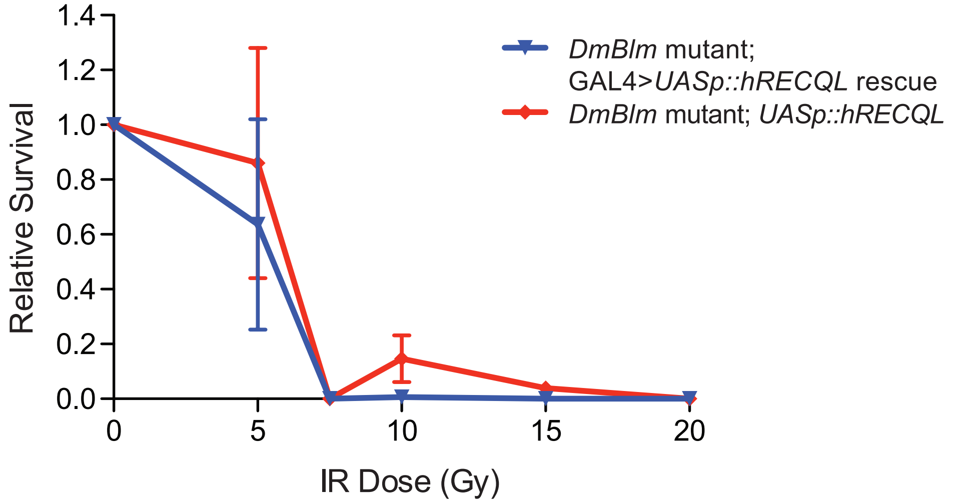

Supplementary Figure S4: Data presented in Figure 5C but separated by female (A) and male (B) survival.
